# Mechanisms and Adaptation Strategies to Improve Heat Tolerance in Rice. A Review

**DOI:** 10.3390/plants8110508

**Published:** 2019-11-15

**Authors:** Shahbaz Khan, Sumera Anwar, M. Yasin Ashraf, Binish Khaliq, Min Sun, Sajid Hussain, Zhi-qiang Gao, Hafeez Noor, Sher Alam

**Affiliations:** 1 College of Agriculture, Shanxi Agricultural University, Taigu 030801, China; shahbazbaloch@mail.hzau.edu.cn (S.K.); sunmin@163.com (M.S.); hafeeznoorbaloch@gmail.com (H.N.); sherjunaid1855@yahoo.com (S.A.); 2 Institute of Molecular Biology and Biotechnology, The University of Lahore, Lahore 54000, Pakistan; niabmyashraf@gmail.com (M.Y.A.); binishafzal@yahoo.com (B.K.); 3 China National Rice Research Institute, Hangzhou 311400, China; sajidhussain@caas.cn

**Keywords:** global warming, rice genotypes, high temperature, breeding, genetic variations, transgenic rice

## Abstract

The incidence of short episodes of high temperature in the most productive rice growing region is a severe threat for sustainable rice production. Screening for heat tolerance and breeding to increase the heat tolerance of rice is major objective in the situation of recent climate change. Replacing sensitive genotypes with heat tolerant cultivars, modification in sowing time, and use of growth regulators are some of the adaptive strategies for the mitigation of yield reduction by climate change. Different strategies could be adopted to enhance the thermos-tolerance of rice by (1) the modification of agronomic practices i.e., adjusting sowing time or selecting early morning flowering cultivars; (2) induction of acclimation by using growth regulators and fertilizers; (3) selecting the genetically heat resistant cultivars by breeding; and, (4) developing genetic modification. Understanding the differences among the genotypes could be exploited for the identification of traits that are responsible for thermo-tolerance for breeding purpose. The selection of cultivars that flowers in early morning before the increase of temperature, and having larger anthers with long basal pore, higher basal dehiscence, and pollen viability could induce higher thermo-tolerance. Furthermore, the high expression of heat shock proteins could impart thermo-tolerance by protecting structural proteins and enzymes. Thus, these traits could be considered for breeding programs to develop resistant cultivars under a changing climate.

## 1. Introduction

Global climate change has resulted in an increase in air temperature by 0.5 °C in the 20th century and temperature is predicted is to be further increased by 1.5 to 4.5 °C in this century [1]. Rice is already growing in areas where temperature has reached optimal for rice growth; therefore, any additional increase in day or night temperature or exposure to high temperature during sensitive stages reduces the rice yields [2]. High temperatures result in serious adverse effects on rice production [3]. It is estimated that the increasing temperature would reduce rice production by 41% at the end of the 21st century. This change can potentially introduce greater sensitivity to previously unaffected developmental stages, such as panicle initiation, spikelet differentiation, gametogenesis, and flowering stage.

Heat stress is commonly defined as an increase in temperature above a threshold level for a certain period that causes irreversible damage to the growth and development of plants [4]. The high temperature at the flowering stage of rice could induce the failure of pollination that is caused by the poor theca dehiscence, increase sterility, and result in a significant reduction of seed number and grain yield [5,6]. The high temperature stress at the grain filling stage could reduce the assimilate supply from shoot to grain, shorten grain filling duration, and ultimately reduce grain yield [7,8]. Heat stress during grain filling also leads to the reduced utilization of additional non-structural carbohydrates in the sink, despite increased assimilate supply from leaves and a reduction in starch metabolism enzymatic activity [9].

The heat tolerance of a plant, is its capacity to produce economical yield and show normal growth under high-temperature conditions by adjusting structural or metabolic properties [10,11]. Traits for heat tolerance are controlled by multiple genes and are related to the morphological and physiological adaptations in rice, whereas little information is available about stress avoidance and tolerance mechanism in rice. 

Heat resistance of a plant can be categorized into avoidance, escape, and tolerance. In the escape mechanism, the plant tries to complete reproduction before the onset of heat stress. In the case of avoidance, the plant maintains high water status by reducing leaf area, closing stomata senescing older leaves under stress. Heat tolerance is conferred by maintaining plant functions and efficient scavenging of reactive oxygen species [12] and stabilizing the structure and function of protein and enzymes by heat shock proteins (HSPs) [13].

Some authors have recently described the adaptation strategies of rice to heat stress [14,15]. Rice escape or avoid the heat by adjusting the time of panicle emergence, spikelet opening, and anther dehiscence relative to the occurrence of heat stress [14]. Spikelet sterility is the most measured trait to access the heat tolerance of rice genotypes and increased by increasing the temperature above a critical limit. There was a 7% reduction in spikelet fertility in IR64 (lowland indica) and 2.4% reduction of spikelet fertility in Azucena (upland japonica) above 33 °C [16]. The variation among genotypes to maintain spikelet fertility under different temperatures could be taken as a tolerance mechanism [17]. Spikelet fertility is considered to be well related to the panicle temperature [15] (Julia and Dingkuhn 2013). One avoidance mechanism is lowering panicle temperature by transpiration cooling [18]. Matsui et al. (2001) reported that plants could decrease the panicle temperature by 10 °C by transpiration, which maintains the spikelet fertility. Similarly, longer and erect top leaves that protect the panicle from direct sunshine also confer heat tolerance [15].

**Figure 1:**
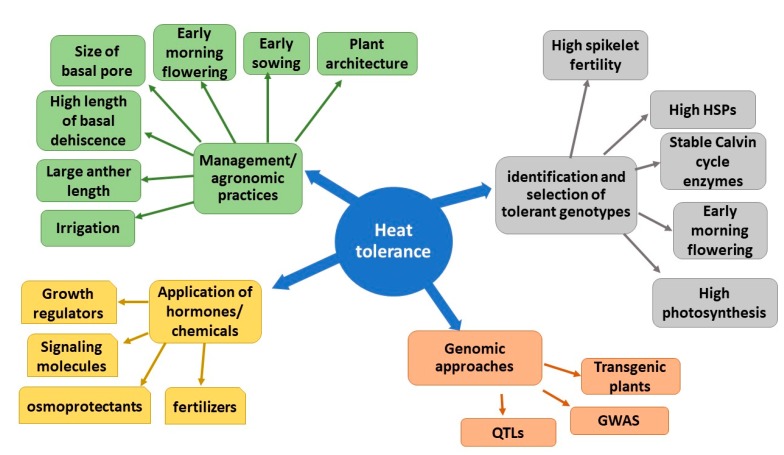
Various approaches to improve heat tolerance in rice.

Coping with heat stress involves different options that account for the ability of plants to escape (early anthesis time), avoid (panicle cooling through transpiration), or tolerate (presence of genes of interest) heat at flowering (Figure 1). Different approaches which could be adopted to increase heat tolerance in rice, are mentioned below.

## 2. Crop Management Practices for Heat Stress Avoidance

### 2.1. Agronomic Management

To cope with high-temperature stress, most of the agronomic management practices focusing on early sowing of rice, adjustments of site-specific cropping and irrigation system, adopting a late or early maturing cultivar to escape high temperature during grain filling [2,19]. The timely sowing of rice varieties is crucial to avoid elevated temperature at the sensitive growth stages. Setiyono et al. [20] conducted a simulation model study in rice fields in two districts of India where the late-planted rice field suffered high yield losses due to the occurrence of heat stress at the reproductive stage and heat-induced spikelet sterility. Therefore, they suggested that the yield losses in these areas could be reduced by the early planting of rice.

The sowing time has an immense role in determining the grain quality of rice due to several environmental factors. Some reports indicated that adjusting sowing time by avoiding heat stress could ameliorate the negative effect of heat stress due to global warming and the deterioration of grain quality of rice. Zhu et al. [21] evaluated the effect of high temperature under different sowing times on grain quality of rice genotypes and reported that the adjustment of sowing time is an efficient management strategy for avoiding heat stress. However, the adjustment of sowing time is difficult, as it also affects the preceding crop and farmers have to plan about the cropping pattern of the whole year.

The release of methane gas under the anaerobic flooded system and nitrous oxide under aerobic condition are the main factors contributing to global warming. Adjustments of irrigation system, such as adopting alternate wet and dry irrigation for aerobic rice, is an alternate solution for decreasing heat stress by reducing the emission of methane [22]. Conserving soil moisture by adding crop residues and manure in soil or modification of microclimate by shading could reduce the temperature to cope with high-temperature stress [2].

### 2.2. Heat Avoidance through Early Morning Flowering

The occurrence of high temperature at anthesis caused spikelet sterility, which resulted in yield reduction in tropical Asia and Africa [23]. Spikelet sterility mainly occurs because the numbers of germinating pollen grains are reduced and anthers fail to dehiscent under high temperature [5]. Even the spikelets of the tolerant cultivars become sterile if exposed under high temperature at the anthesis stage. At the anthesis stage, the complete sterility of anthers was recorded in tolerant cultivar ‘N22’ when exposed to 41 °C [24], whereas half of the spikelets of tolerant japonica cultivar ‘Akitakomachi’ were found to be sterile when exposed to 40 °C [25]. The anthesis is the most sensitive stage, because the high temperature most affects spikelet fertility at the time of flower opening. Moreover, only exposure of one hour of high temperature induces spikelet sterility [16], however, high-temperature treatment after 1 h of flowering does not induce sterility, indicating that spikelets become tolerant to high-temperature stress after fertilization [24]. Other studies also confirmed that only one hour of high temperature or less is sufficient to induce sterility [16,26]. Thus, the timing of anthesis has significant importance for phenotyping for high-temperature tolerance in term of spikelet fertility.

Genotypic variation exists in rice cultivars for anthesis time. Early flowering could be considered as an escape mechanism for avoiding elevated temperature stress [14,27]. Flower opening time varies with the variety, for instance, the flower opening time of *Oryza glaberrima* occurs before than *Oryza sativa* and their interspecific hybrids [28,29]. Similarly, the flower opening time of *Oryza sativa* cultivars is mostly 1–2 h earlier than in Koshihikari’ which is a standard Japanese cultivar [30]. In indica cultivars of rice, flower opens almost 45 min earlier to that time when temperatures rise to 7 °C more than the normal air temperature [16]. 

Early morning flowering is a useful trait that could be used to reduce spikelet sterility by advancing the time of flowering in the early morning when the air temperature is cooler [31]. The identification of genes responsible for controlling the flowering time is essential for determining the tolerance mechanism and imparting heat tolerance. Early flowering trait could also be exploited in breeding to impart heat tolerance to rice genotypes in the flooded rice system [32,33]. Ishimaru et al. [23] transferred the early flowering trait from *Oryza officinalis* (a wild rice) into *Oryza sativa* cultivar ‘Koshihikari’. The panicle opening of introgression line expressing early-morning flowering trait was a few hours before Koshihikari when the temperature was cooler. When the spikelets received the steadily rising temperature according to the daytime change, and earlier flower opening then decreased the spikelet sterility in the introgression line. However, when the panicles were exposed to high temperature in chamber (38 °C) at anthesis, then both lines showed 60% spikelet sterility, demonstrating that high-temperature tolerance was similar in both genotypes [23].

Recently, Bheemanahalli et al. [33] assessed the flowering pattern (first spikelet opening and peak spikelet opening time) of 289 rice cultivars to determine the agronomic importance of this trait. This was the first study that records the key flowering pattern traits among a diverse set of cultivars from tropical and subtropical countries originating from 13 tropical and 20 subtropical countries. Significant variation existed among cultivars for the studied flowering traits and spikelet sterility. First spikelet opening time after dawn was varied from 2.35–5.08 h in the dry season and 3.05–5.50 h in the wet season, while the peak spikelet opening time ranged between 3.32–6.27 h in the dry and 3.50–7.05 h in the wet season. In the near-isogenic line (IR64 + qEMF3), spikelet sterility was effectively reduced by 71% during dry seasons in the hot tropical climate as compared to the tropical and subtropical cultivars. No other subtropical and tropical cultivar and introgressed line (IR64 and Nanjing) possessed traits for early morning flowering, which indicated the usefulness of these traits for overcoming high temperature. Introgression of quantitative trait locus (QTL) for early morning flowering trait in the indica genetic background (IR64 + qEMF3) shifted the peak anthesis by almost 2.0 h than in recurrent parent (IR64), and effectively reduced the spikelet sterility under high temperature [34]. However, these studies were conducted in the subtropical climate, its agronomic effect and stability of these traits have not yet been studied under tropical climate.

Flower opening time is a genetically controlled factor; however, it is also affected by other external factors, like air temperature, humidity, and solar radiation [35]. Some previous studies indicated that the flower opening time could be controlled by dark treatment, day and night temperature, or by hormones. Nishiyama and Blanco [36] found that the flower opening time could be delayed up to two hours by one-hour dark treatments to flowers. Kobayasi et al. [35] suggested that rice plants might open their flowers earlier by detecting the higher night temperature. The difference in day and night temperature also controls the flower opening time and 29/21 °C day and night temperature advanced the flower opening than 25/25 °C. However, these techniques are impractical to adopt in the field or extremely expensive. 

### 2.3. Size of Basal Pore 

Basal pores open at anthesis when the anthers become erect in position. Basal pore length varies with the rice cultivars. A larger length of basal pore facilitated the release of pollen grains from basal pore to the stigmata during anther dehiscence; therefore, the number of pollen grains on stigmata is related to the length of the basal pore [37]. In contrast to which in case of a small basal pore, most of the pollen grains remain inside anthers until floret opening after which the anthers bend and pollens are dispersed by wind. Therefore, varieties with small basal pore make self-pollination unpredictable and are more likely are cross-pollinated [38]. The larger size of basal pore facilitated the release of pollens from anthers and thus the chances of pollination also increased. As this trait is easily recognized, it could be used as a screening tool for heat tolerance and breeding.

### 2.4. Anther Size

The length of anther varies with genotypes and genotypes with larger anthers are relatively more tolerant to temperature stress at the booting and flowering stages [5]. Under temperature stress, floret sterility is the direct cause of less pollen grains being germinated on the stigma due to poor anther dehiscence [24]. Anther size is positively correlated with the number of pollen grains per anther; therefore, it has been assumed that cultivars with larger anthers have a higher number of pollen grains, which compensates for the temperature-induced decline in the number of germinated pollen grains [5].

### 2.5. Length of Basal Dehiscence of Anther

Proper dehiscence of the anther is necessary for the release of pollen grains to enable pollination and fertilization. Certain developmental events are required for successful dehiscence, including swelling of pollen grains and increasing pressure break the septum [39]. Under high-temperature stress, the theca dehiscence is affected due to the inability of swelling of pollen grains and pollen release [37,40]. Longer basal dehiscence will increase the chances that the pollen grains have been transported to the stigmata [37,38]. Therefore, the genetic improvement of rice genotypes with larger basal dehiscence will increase pollen shedding and will be helpful in compensating for the decrease in pollen swelling and theca dehiscence under high temperature. Furthermore, the length of basal dehiscence is easy to measure and remains stable under normal and high temperature condition, and thereby could be used as a morphological marker to measure the tolerance of rice genotype to high temperature [37,38]. 

### 2.6. Plant Architecture

Developing and selecting genotypes having a suitable architecture might be helpful for imparting high-temperature tolerance. Genotypes will be more heat tolerant if their panicles are surrounded by a higher number of leaves that will protect and shade the anther, create cooling by transpiration, and prevent anther transpiration [41]. The less transpiration from anther will ensure the swelling of the pollen grains, which is essential for anther dehiscence. It has been reported that the plant height of N22 and its mutant NH219 was increased under high temperature, which also increased thermo-tolerance [42]. Increasing plant height will also increase the transpiration rate, which will help in the avoidance of high-temperature stress. It has also been reported that varieties with asynchronous tillers and panicle development showed less yield reduction and they were relatively tolerant of heat stress at a critical developmental stage. In such varieties with asynchronous panicles, all of the panicles are not exposed to high temperature at their critical stage and could escape heat stress [31]. However, the breeders did not select such varieties due to the longer ripening period and yield loss due to other factors.

## 3. Induction of Acclimation by Using Growth Regulators/Protectants/Chemicals

### 3.1. Growth Regulators

Salicylic acid is a phytohormone with ubiquitous distribution among plants and it regulates a variety of physiological processes in plants to abiotic stresses [43]. Mohammad and Tarpley [44] stated that the exogenous application of salicylic acid on rice alleviated the adverse effect of a high night temperature of 32 °C by increasing dry matter partitioning up to 16%. The foliar application of salicylic acid (0.1 mM) induced high-temperature tolerance and ameliorated the effect of heat stress in rice [45]. Similarly, Zhang et al. [46] reported that the foliar spray of salicylic acid (0–50 mmol L^−1^) alleviated the adverse effect of heat stress by enhancing the proline, sugar, antioxidative enzymes, such as POD, APX, and CAT, and phytohormones concentrations, like GA3, IAA, and ABA contents of rice spikelet ultimately increased the yield, seed setting rate, and spikelet numbers. Chang et al. [47] reported that Class II HSP (*Oshsp18.0*) was induced by heat shock and salicylic acid treatments in rice, which indicates the role of salicylic acid in the induction of plant response to stress.

Some growth regulators, such as methyl jasmonate, which advances the flowering time to early morning, could be used to mitigate heat stress [35]. Kobayasi and Atsuta [48] conducted an experiment to study the effect of methyl jasmonate on the flower opening time and reported that the application of methyl jasmonate advances the flower opening time two hours earliest, however it also increased the number of flowers that opened after treatment, which otherwise should have to open the next day in the absence of treatment and resulted in spikelet sterility due to poor pollination and indehiscence. The flower opening was initiated just 80 min after the application of methyl jasmonate [49].

Ascorbic acid (vitamin C) plays various important roles in plants, such as regulating cell division and elongation, serving as a coenzyme to scavenge ROS as an antioxidant, in the biosynthesis of organic acids, phytohormones, and falvonoids [50]. Endogenous ascorbic acid protects plants from oxidative stress and maintains the stability of photosynthetic function. Zhang et al. [51] studied the effect of endogenous ascorbic acid content by using transgenic rice with overexpressed and suppressed GLDH (L-galactono-1,4,-lactone dehydrogenase) enzyme activity under high temperature. Higher ROS were found in transgenic rice with suppressed GLDH. Endogenous ascorbic acid reduced the degradation of Rubisco and chlorophyll, and the accumulation of ROS thus imparts stability to rice under high temperature. 

The alpha-tocopherol also acts as an antioxidant is one of the most effective single-oxygen quenchers. Mohammed and Tarpley [52] found that japonica rice plants that were sprayed with 2.3 kg ha^−1^ of α-tocopherol showed 6% increases in grain yield by increasing membrane integrity and spikelet fertility and decreasing respiration. They reported a possible role of α-tocopherol in membrane stability with less generation of reactive oxygen species. Increased membrane stability also decreased the respiration rate, because, under oxidative stress, the plant increases maintenance respiration to sustain repair mechanisms of damaged membranes. 

Exogenous application of brassinosteroids regulates signal transduction pathways by enhancing the biosynthesis of endogenous hormones, such as brassinosteroids, zeatin riboside, indole-3-acetic acid, jasmonic acid, and gibberellic acid, and stimulates stress tolerance [53]. Previous reports indicated that, under high-temperature stress, application of brassinosteroids induced thermal tolerance by increasing the synthesis of HSP, and also by increasing expression of genes for protective enzymes [54,55]. Chandrakala et al. (2013) [45] investigated the effect of foliar application of 24-epi-brassinolide on the physiology of rice cultivars (Pusa Sugandh 5 and Nerica L 44) that were grown under ambient and high temperature (36 °C) environments. They found a strong positive relationship between grain yield and leaf photosynthesis by the pre-treatment of 24-epi-brassinolide. All three studied concentrations of 24-epi-brassinolide (0.5, 1 and 1.5 ppm) ameliorated heat stress, as indicated by improved photosynthetic activity. Sonjaroon et al. [55] had foliarly applied epibrassinolide (brassinosteroid) and two brassinosteroid mimics (having the same chemical structure and function similar to brassinosteroid) at the reproductive stage to check the physiological response of rice under high temperature. They reported that these hormones and hormone mimics were effective in enhancing the heat tolerance in rice by increasing the net photosynthesis, transpiration rate, and stomatal conductance.

Auxin plays a role in maintaining spikelet fertility and a reduced level of active IAA could cause pollen abortion, which is a common reason for male sterility. Under high temperature the level of auxin reduced and reduction of IAA and GAs was more in heat susceptible cultivar than tolerant genotype [56]. Zhang et al. [51] studied the effect of spraying auxin on the elongation of pollen tubes of heat-tolerant and susceptible genotypes. They reported that spraying naphthaleneacetic acid reduced and reversed the spikelet sterility of both the heat susceptible and tolerant genotypes of rice by inhibiting the reduction of pollen tube growth.

Fahad et al. [57] tested different combinations of plant growth regulators (PGR) on the growth and yield of two rice cultivars grown high day and night temperature and concluded that the exogenous application of combination of PGR (ascorbic acid, alpha-tocopherol, methyl jasmonate, and brassinosteroid) augmented heat tolerance, as indicated by high photosynthesis rate, water use efficiency, and increased yield. The combination of vitamin C, vitamin E, methyl jasmonates, and brassinosteroids has improved the grain yield by 27–63% and 15–13.6% under high day and high night temperatures, respectively. Similarly, the combined application of salicylic acid, alpha tocopherol, and glycine betaine improved the tolerance of rice plants to high-temperature stress and increased yield [44].

### 3.2. Use of Organic Elicitors, Fertilizers, or Signaling Molecules

The application of CaCl_2_ (10 mM) ameliorated the heat stress-induced reduction in gas exchange, PSII efficiency, photosynthetic water use efficiency, spikelet fertility, and leaf chlorophyll content in rice [45]. Shahid et al. [58] reported that the application of boron in soil was effective in ameliorating the negative effects of heat stress, and boron applied rice plants showed improved cell membrane stability and spikelet fertility and higher yield. 

Nitric oxide role as signaling molecule regulates many developmental processes in plants, including flowering and fertilization and tolerance against high-temperature stress [59]. Uchida et al. [60] reported that rice seedlings that were pretreated with low levels of hydrogen peroxide or nitric oxide showed higher heat tolerance with less senescence and high PSII quantum yield. Low doses of these signaling molecules activated the antioxidative system and also the expression of heat stress-responsive genes, such as HSP gene and sucrose phosphate synthase gene, were higher in pretreated seedlings.

### 3.3. Use of Osmoprotectants

The accumulation of osmoprotectants is an important adaptive mechanism of plants in response to various types of abiotic stresses, including high temperatures [61]. In spite of the role in cytoplasmic osmotica, they also protect the metabolic processes by stabilizing membrane, oxygen-evolving photosystem complex, and activities of various enzymes. 

Glycine betaine is an important compatible solute, which is accumulated in plants under high temperature [62] and imparts heat tolerance by protecting Rubisco and citrate synthase and other enzymes from heat degradation [63]. Some plant species, such as sugarcane and maize, can accumulate high concentrations of glycine betaine under high temperature, whereas, rice, arabidopsis, mustard, and many other plants could not accumulate glycine betaine [64,65]. Therefore, exogenously applied glycine betaine increased yields under high temperatures, possibly acting through previously observed increased antioxidant levels, which might have protected the enzymes and membranes from degradation [44]. The addition of glycine betaine protects the thermal degradation of the rubisco enzyme in the leaves of rice seedlings at temperature stress of 35 to 45 °C [66]. Glycine betaine increases the percent pollen germination and spikelet fertility in rice and it will be also useful in mitigating the yield reduction threats. Proline protects the thermal degradation of the rubisco enzyme in rice seedlings at high-temperature stress [66].

Spermidine is a polyamine and it has been reported to play an important role in tolerance of various stresses, including heat stress [67]. Tolerance to heat by spermidine application is mainly attributed to the induction of antioxidative enzyme activities, and starch and polyamine metabolism. Starch synthesis enzymes expression was increased in rice seeds after the exogenous application of spermidine [68]. Spermidine treated plants showed reduced hydrogen peroxide and propionaldehyde contents. The exogenous application of spermidine also modulates the level of glutathione and glyoxalase system [69]. Tang et al. [68] indicated that spraying spermidine after flowering had alleviated the adverse effect of high temperature on the yield of japonica rice. The spermidine treated plants showed higher grain filling rate, grain yield, and antioxidative enzymes, whereas reduced malondialdehyde (MDA) accumulation than without spermidine. Application of spermidine also increased soluble sugars content, photosystem II (FPSII), photosynthetic electron transport rate (ETR), photochemical reaction of light energy ratio (Pr), variable fluorescence/maximum fluorescence ratio (Fv 0/Fm 0), stomatal conductance, and ultimately improved the photosynthesis and transpiration rate [68]. Fu et al. [70] reported that exogenously applied spermidine increased rice tolerance to heat by modulating the antioxidants, polyamine metabolism, and starch accumulation. 

## 4. Breeding Approaches by Identification and Selection of Heat-Tolerant Genotypes 

A wide variability among rice genotypes exists in response to resist high temperature. Genetic resistance for heat stress is defined as when the growth and production of some genotypes are less affected than other genotypes under heat stress. Therefore, replacing the sensitive genotypes with tolerant one will maintain the production of rice, even under the occurrence of high temperature [41,71].

Different response of rice cultivars to high temperature indicates the possibility to investigate genotypes that are better adaptable to high temperature. Genetic variability could be utilized to screen tolerant genotypes and could be utilized for developing tolerant and well-adapted cultivars in a future warmer climate. Screening of rice cultivars is going on in some Asian countries. For instance, Masuduzzaman et al. [72] had studied 1217 rice germplasms from different rice-growing areas with a hot climate and found that only 2% of the genotypes presented a certain extent of thermal tolerance. IR 87606–109–2–2 and IR 86991–146–2–1–1 were found as tolerant to heat during flowering. The identified germplasms with heat tolerance could be studied for understanding the heat tolerance mechanisms and used for improving the heat tolerance of future rice genotypes. However, genotypes that are tolerant to one kind of high temperature stress might not be necessarily tolerant of other kinds of heat stress [73].

### 4.1. Low Leaf Temperature and Panicle Temperature and Well Exerted Panicle

Some genetic variability was reported in the cooling capacity of the leaf and panicle due to differences in the transpiration rate [18]. Heat stress appears to be more damaging to the panicles when compared to leaves, because the transpiration rate in the flag leaf is higher than in the spikelet under heat stress resulting in less leaf temperature than the spikelets [74]. Spikelet sterility has been found to be correlated to panicle temperature [75]. The flag leaf temperature of the tolerant (N22) cultivar was found to be less as compared to the sensitive cultivar (GT937) at high-temperature treatments [74]. Jumiatun et al. [76] conducted a study in Indonesia and concluded that rice cultivars that maintain low leaf temperature and had well-exerted panicle showed better ability to tolerate high temperatures with a high percentage of filled grain. The leaf temperature of IR64 is low, whereas, Jatiluhur and Menthik Wangi had well-exerted panicle, but still have a low percentage of filled grain. Thus, increasing spikelet fertility by cooling effect provides a new opportunity to increase heat tolerance. However, the cooling ability by transpiration is less successful under humid conditions [15].

### 4.2. High Carbohydrate Availability and Photosynthetic Rate

One approach to achieve high thermal tolerance is to screen out the genotypes that could maintain a higher non-structural carbohydrate pool higher biomass and stable photosynthesis under high temperature and these genotypes could serve as genetic donors for reducing temperature-induced yield loss. Heat tolerant genotypes could maintain photosynthetic activity for a longer time after anthesis and thus could produce higher grain weights. After anthesis or the reproductive stage, photosynthesis is the second most susceptible factor to heat stress. Therefore, preservation of the photosynthetic rate is critical to achieve thermo-tolerance and maintain normal growth [77]. The temperature response of gas exchange traits was investigated in the field while using rice genotypes, IR20, IR53, IR46, and N22 at high day and night temperature in a phytotron. It was found that all photosynthetic traits of N22 were higher than the other genotypes at elevated temperature, indicating a higher tolerance of N22. The photosynthesis rate was first increased with temperature up to an optimal (32 °C), which then decreased by increasing temperature up to 42 °C [78]. 

### 4.3. Protection from Thermal Degradation of Calvin Cycle Enzymes 

Rubisco (ribulose 1–5, bisphosphate carboxylase oxygenase) protein is extremely temperature-sensitive and it degrades due to photosynthetic capacity becoming extremely limiting under high temperature [79]. Genetic differences exist in rice genotypes regarding the degradation of Rubisco under high temperature [80]. Scafaro et al. [81] ascribed the higher tolerance of wild rice (*Oryza meridionalis*) to the increased abundance of Rubisco after at elevated temperature. Transgenic rice that overexpressed Rubisco activase enzyme could maintain higher photosynthetic activity under heat stress [82]. Heat tolerant genotypes showed comparatively higher expression of various Calvin cycle enzymes (Rubisco activase, and phosphoribulokinase) under high-temperature stress. Rubisco activase enzyme is responsible for the activation of Rubisco and phosphoribulokinase is responsible for the regeneration of Rubisco in the final step [80,83]. Rubisco activase is highly susceptible to high temperature, but rubisco activase of heat-tolerant wild rice (*Oryza meridionalis*) is relatively tolerant to high temperature (up to 40 °C) when compared to *Oryza sativa* [80]. Recently, Scafaro et al. [84] designed an experiment to study the heat tolerance of recombinant lines overexpressing rubisco activase gene from *Oryza australiensis* and reported higher thermal tolerance of variants of rubisco activase with high growth and seed yield. The regeneration of Rubisco also altered by high temperature and presence of a large pool of Rubisco subunit in heat-tolerant genotypes (NH219) also compensates the temperature-induced deactivation of Rubisco [85].

### 4.4. High Production of Heat Shock Proteins

The higher gene expression belonging to heat shock protein factors (Hspf) and heat shock transcription factors (Hsfs) is considered to be important in the plant’s response to heat stress [86]. HSPs are important molecular chaperones that assist the assembly and folding of protein, prevent irreversible aggregation and degradation of misfolded protein to maintain cellular homeostasis under both optimal and adverse developmental conditions [73]. Jagadish et al. [87] studied the protein expression changes in anther under heat stress and identified that the heat-tolerant variety (N22) expressed more HSPs under heat stress, which contributed to heat tolerance with high spikelet fertility when compared to heat-sensitive Morobrekan with 18% spikelet fertility. Therefore, it is speculated that HSPs induce higher tolerance to heat stress in rice. 

Gene expression analysis indicated significant variation in the timing and level of gene expression encoding Hsp and Hsf in the leaves of different rice genotypes. Heat tolerant genotypes (N22 and RF-42, R-1389) showed a higher level of expression for most of the tested HSPs and Hsfs [88]. Later on, one more study indicated that various Hsfs, for instance, OsHsfA7, OsHsfA2a, and OsHsfA2e were extremely upregulated in N22 under heat stress of 42 °C imposed during flowering initiation stage. Upregulation in these transcription factors were 17, 49, and 6-fold increased as compared to normal temperature. The expression of Hsfs (OsHsfA2e and OsHsfA7) was also up-regulated in Vandana, but the increase in expression was very low when compared to N22 [89]. 

Lin et al. [73] studied the heat acclimation memory of japonica (Nipponbare) and indica (N22) rice genotypes. Both of the genotypes showed a contrary response for basal tolerance and long term acquired tolerance to heat stress and decay of HSPs. N22 showed faster decay of HSP101 than the japonica rice, which might be attributed to ecological modification. A positive feedback loop was found at the posttranscriptional level between two heat-inducible genes, HSP101 (HSP family 100) and HSA32 (heat stress-associated 32-KD protein), which extend the effect of heat acclimation. Nipponbare has more long term acquired thermal tolerance, whereas N22 has greater basal thermal tolerance [90]. 

### 4.5. Higher Cell Membrane Thermostability and Chlorophyll Fluorescence

Genotypic variation has been documented in rice for chlorophyll fluorescence parameters under heat stress [89]. Tolerant genotype N22 has been shown to maintain a high Fv/Fm ratio under heat stress of 42.2 °C [91]. High temperature reduced the chlorophyll content and the reduction was more in thermo-sensitive genotypes [92]. According to Sailaja et al. [89], membrane thermostability was the most reliable trait that could be used to screen out the tolerant rice genotype and it showed a higher correlation with yield under high temperature. High-temperature stress increased the mean relative injury and higher relative injury was observed in the BPT5204 (90%), followed by Swarna (80%), Krishna Hamsa (42.4%), and Vandana (43%), whereas, less relative injury was observed in the four tolerant genotypes, which also showed less reduction in yield at high temperature. A reduction in rice yields as a result of high night temperature has been attributed to increased leaf electrolytic leakage [44].

Lian et al. [93] studied the effects of high temperature on physiological and biochemical characteristics in the flag leaf of rice during the heading and flowering period. The results showed that the membrane permeability in high-temperature sensitive line 4628 was more significant under high-temperature stress than in high-temperature tolerant line 996. Maavimani et al. [94] determined the genetic variation among genotypes for MTS and RI and their relation with yield attributing traits. A total of 48 rice recombinant inbred lines along with their parents derived from the cross between IR 64 and N 22 (heat tolerant) were utilized for this study. The estimates of phenotypic and genotypic coefficients of variations were high for all of the traits, except plant height, relative injury, and panicle length. Membrane thermostability had a positive and significant correlation with grain yield per plant. Path analysis revealed that membrane thermostability had a direct effect on grain yield per plant and emphasized that selection will be effective through this trait in heat-stressed environments.

### 4.6. Anther Dehiscence, Spikelet Fertility, and Yield Attributes

Genetic variation exists for the anther and spikelet sensitivity to elevated temperature. The anthers of heat-tolerant rice genotype dehiscence more easily than those of the susceptible ones at high temperature [71,87]. Spikelet fertility and yield per plant are two important criterions that could be used to access heat tolerance. The most tolerant rice accessions identified under heat stress is N22, which maintained high spikelet fertility of 71%, even when it was subjected to 38 °C for 6 h at the anthesis stage, while the spikelet fertility of moderate tolerant indica accession IR64 was 48% and in the japonica variety Moroberekan was 18% [87]. However, Prasanth et al. [95] reported that, without considering yield per plant, spikelet fertility alone is not a reliable criterion to access heat tolerance. They determined that heat tolerance in rice lines developed from mutant and wild species and found that rice line S-65 and S-70 showed low spikelet fertility and high yield per plant consistently in response to heat stress. 

### 4.7. Breeding

Modern researchers are using double haploid, recombinant inbred line, and backcross inbred lines that were obtained from crosses between tolerant and sensitive cultivars, mostly developed by crosses between indica and japonica or indica and indica cultivars, to scrutinize the genetic basis of rice for the heat tolerance at different developmental stages [96,97]. Introgression line obtained from hybridization between wild rice (*Oryza officinalis*) and indica cultivar Koshihikari (*Oryza sativa*), expressing early morning flowering trait showed higher spikelet fertility and yield under heat stress as compared to the late flower opening genotype [23].

**Figure 2:**
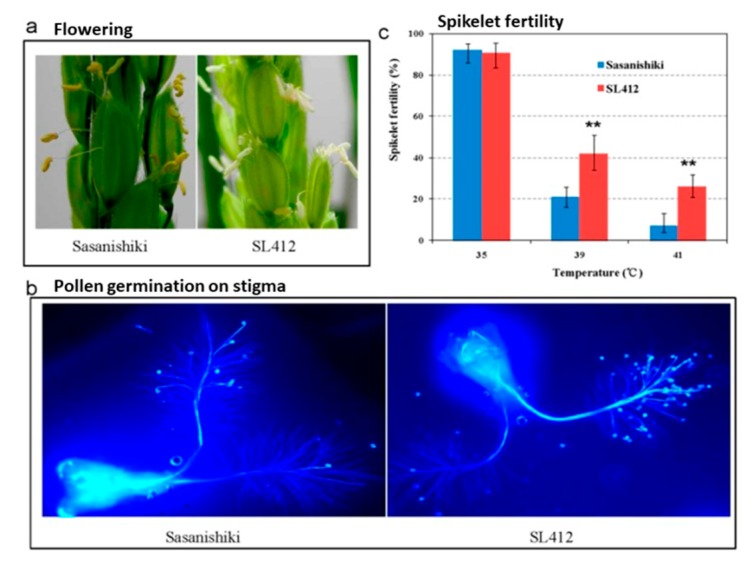
Heat tolerance of heat-sensitive japonica (Sasanishiki) and tolerant CSSL line (SL412) at high temperature showing the (**a**) flowering, (**b**) pollen germination, and (**c)** spikelet fertility [107].

Genetic studies showed that the heat tolerance of rice is a multigenic trait, being controlled by diverse sets of genes and varies with the development stages and tissues of plant [98,99]. With the advancement of molecular marker technology, the detection of QTLs for conferring heat tolerance and investigation of its genetic effects has become possible by identifying the loci that are linked to heat tolerance. The detection of QTLs helped in understanding the genetic mechanism, marker-assisted selection, and QTL cloning in rice. Various experiments have been conducted for the identification of QTLs for heat stress [97,100,101,102,103]. Most of the QTLs for heat stress tolerance were identified during the flowering stage. QTL linked with traits, like spikelet sterility, yield, and stay green, were mapped mostly on chromosome 1, 4, 7, 9, and 10 on all 12 linkage groups [6,96,97,100,101,103,104,105,106,107]. Zhao et al. [107] used chromosome segment substitution lines (CSSL) obtained from heat tolerant indica (Habataki) and heat susceptible japonica (Sasanishiki) genotypes. They identified QTLs that were related with spikelet fertility (*qSFht2* and *qSFht4.2*) and flowering time (*qDFT3*, *qDFT8*, *qDFT10.1*, and *qDFT11*). Amongst the obtained CSSL, especially SL412 showed significantly higher spikelet fertility than Sasanishiki and 6 CSSL showed higher pollen shedding level (Figure 2).

Zhang et al. [101] identified two SSR (simple sequence repeats) markers (RM3586 and RM3735) that were responsible for 3% and 17% of the variation for high-temperature stress tolerance and suggested that these genetic loci could be used in marker-assisted selection for heat tolerance breeding. After this study, Jagadish et al. [95] crossed a susceptible (Azucena) and tolerant (Bala) genotypes and studied F6 population of a recombinant inbred line. They recognized eight QTLs that were linked with spikelet fertility under heat on the different chromosomes. Most significant high temperature-responsive QTL, responsible for 18% phenotypic variation, was mapped on chromosome 1. Xiao et al. [102] used the pollen sterility trait as an indicator of heat tolerance and identified two QTL_S_ (*qPF4 and qPF6*), which increased the pollen fertility in rice under heat stress. Xiao et al. [102] and Ye et al. [108,109] identified several QTLs showing variation in spikelet fertility under high temperature. 

A QTL for early flower opening trait (qEMF3) was detected in wild rice (*Oryza officinalis*) was used to develop the near-isogenic line of indica rice and shifted the flower opening time to 1.5–2.0 h earlier in cultivar Nanjing 11 and IR64, which were resistant to heat stress at the flowering stage [34]. Shanmugavadivel et al. [106] identified QTLs for heat tolerance at the flowering stage by crossing heat-tolerant genotype (Nagina22) and heat-sensitive indica (IR64).

Huang et al. [110] examined 950 genotypes, including sup-species of indica and japonica cultivars, and identified 32 new loci linked with the timing of flowering. The genome-wide association study (GWAS) method was conducted by Lafarge et al. [111] to detect QTLs for maintaining spikelet fertility under high temperature, for which 167 indica accessions were genotyped with 13,162 SNPs. Fourteen loci were detected, being associated with spikelet sterility from which eight overlapped with QTLS from previous literature. Gene families at loci associated with spikelet sterility were related to plant response to abiotic stress by regulating HSPs and osmotic adjustment. Analysis of diversity at loci associated with spikelet sterility showed a widespread distribution of the favorable alleles in the genetic groups of *Oryza sativa*. Only a few accessions have accumulated favorable alleles at all loci. N22 and some Indian and Taiwanese varieties are effective donors for heat tolerance [111].

## 5. Genetic Manipulations for Heat Tolerant Transgenic Rice

Multiple genes are involved in the synthesis of HSP, which switched on underexposure of high temperature and have an essential role in recovery from heat stress [112,113]. Manipulations of HSPs in transgenic plants have the potential to improve heat stress tolerance and they have a significant impact on the exploitation of the inherent genetic potential of rice [114]. Genetic improvement of rice genotypes for thermotolerance is a feasible practice for rice production under changing climate [114]. However, there are only a few studies reporting heat tolerance by using transgenic rice (Table 1).

Transgenic plants over-expressing *Hsps* have been reported to show increased thermotolerance [90]. The higher acquired tolerance of transgenic lines is attributed to the higher accumulation and expression of levels of HSP chaperones. Experimental evidence from mutant and transgenic species confirmed the role of HSPs in imparting heat tolerance [123]. In rice, Katiyar-Agarwal et al. [90] transformed the HSP (AtHSP101) cDNA from *Arabidopsis thaliana* into the indica cultivar of rice *Pusa basmati* 1. The survival and growth of T2 transgenic lines was improved under high-temperature stress as compared with untransformed plants. Thermo-tolerance was attained in transformed plants due to the overexpression of HSP. Transgenic rice cultivar ‘Hoshinoyume’ overexpressing HSP (sHSP17.7) showed a higher tolerance to heat stress [117]. 

Qi et al. [116] reported that transgenic rice overexpressing mitochondrial gene for mtHsp70 showed higher heat tolerance, as indicated by less program cell death by recovering mitochondrial membrane potential and preventing ROS. The *WRKY* genes are known to encode a large number of transcription factors and they participate in various abiotic stresses. Wu et al. [121] fused the cDNA of *OsWRKY11* to the promoter of HSP101 and introduced it into rice and the plants were exposed to higher temperatures. Transgenic rice overexpressing *WRKY* gene showed increased heat tolerance, as indicated by less reduction of growth traits, higher survival, and desiccation tolerance. Proteomic studies could lead to a better understanding of the molecular basis of heat tolerance of rice plants. Lee et al. [124] investigated proteome in rice leaves under heat stress and reported that almost 73 proteins of low molecular weight were differently expressed, which were mainly related to HSPs, regulatory proteins, and proteins for energy and metabolism. 

## 6. Conclusions and Future Prospective

In the past, there were occurrences of periods of destructively high temperature perhaps once every century. It is predicted that such incidences of sudden high temperature will be increased in the future risking the shortage of food. Therefore, losses in rice production could be avoided by identifying the management techniques, selecting tolerant genotypes, and breeding of suitable rice cultivars. Hence, there is an urgent need to elucidate the physiological and genetic mechanism and develop heat-tolerant varieties for improving rice quality, quantity, and stability of rice production across wide environmental conditions. The adaptation of tolerant genotypes of rice cultivars will also reduce the yield losses. However, care should be taken so that the yield or the qualitative traits should not be suffered by improving plant tolerance to high-temperature stress. High temperature has a negative impact on all stages of rice growth; however, the anthesis stage is the most susceptible stage, at which a mild increase in temperature might significantly reduce the yield. Other noticeable effects of high temperature are pollen sterility, less assimilate partitioning, affected grain filling, structural changes of cell organelles, oxidative stress, lipid peroxidation of cell membranes, disruption of leaf water relations, and reduction in photosynthesis. The acclimation of plants by pre-activation of defense machinery by priming with prior mild heat exposure or any external eliciting agent holds promise to minimize damage, particularly at the sensitive growth stages. Concurrently, applications of exogenous osmoprotectant, plant hormones, and inorganic elicitor might be used to induce a short term acclimation response. Such priming or exogenous applications have been shown to be useful for mitigating high-temperature stress effects specifically when applied at a critical growth stage. However, the efficacy of using these regulators under heat stress needs more studies, because only a few reports are available regarding growth regulators. Genetic variability of rice for heat tolerance could be used for screening germplasm and selecting cultivars that were less affected by the heat stress by opening flowers earlier in the morning and by maintaining spikelet fertility, cell membrane thermostability, chlorophyll fluorescence, HSPs, and maintain a greater non-structural carbohydrate pool with less affected photosynthesis rate when grown in warm environments. 

In the future, efforts are needed to fully explore the genetic diversity by identifying the physiological pathways to maintain sustainable production of rice in the warming climate and evaluate the management options against heat stress. A challenge for the future will be to unravel how these different levels of control are integrated to achieve a robust thermo-tolerance. Now, a collection of strategies, including molecular techniques like genomics, proteomics, and transcriptomics, are needed for the adaptation and implementation and use of a combination of modern approaches, like climatology tools and GIS, are also required. However, a particular genotype is only tolerant to certain conditions, so that trait dependency and variation must be carefully considered.

## Figures and Tables

**Table 1:** Transgenic rice and gene in response to heat tolerance.

Rice Trans Host	Gene	Encoding Protein	Source	Mechanism	Reference
Hoshinoyume	*sHSP17.7*	HSP17.7	*Oryza sativa* L.	CaMV 35S promotor; enhanced heat and drought stress	[115]
Pusa basmati	*AtHsp101*	HSP101	*Arabidopsis thaliana*	CaMV 35S promotor, enhanced heat tolerance	[90]
Nipponbare	*mtHsp70*	HSP70	*Oryza sativa* L.	CaMV 35S promoter; mtHsp70 over-expression suppressed programmed cell death and ROS	[116]
Hoshinoyume	*sHsp17.7*	HSP17.7	*Oryza sativa* L.	CaMV 35S promoter, enhanced heat and UV-B tolerance	[117]
Spl7 mutant	*Spl7*	HSFA4d	*Oryza sativa* L.	CaMV 35S promoter	[10]
*Oryza sativa*	*fad7*	Omega 3, fatty acid desaturase	*Arabidopsis thaliana*	Maize Ubi1 promoter; silencing of endogenous FAD genes	[118]
Zhonghua11 *Oryza sativa* L.	*SBPase*	SBPase	*Oryza sativa* L.	ubiquitin promoter, over-expressing SBPase increased tolerance	[119]
*Oryza sativa ssp. Indica*	*RCA*	Rubisco activase	*Oryza australiensis*	overexpression improved growth and yield	[84]
*Oryza sativa L.*	*rbcS*		*Oryza sativa* L. cv Notohikari	Increased rubisco and photosynthesis in rbcS-sense lines compared to wild type	[83]
Dongjin	*OsGSK1*	Glycogen synthase kinase3-like	*Oryza sativa* L.	enhanced tolerance	[120]
Sasanishiki	*OsWRKY11*	WRKY11	*Oryza sativa* L. *cv. Nipponbare*	HSP101 promoter, increased desiccation tolerance and survival rate of green parts	[121]
*Oryza sativa* L.	*DPB3-1*	DPB3	*Arabidopsis thaliana*	DPB31 overexpression, heat stress inducible genes were upregulated	[122]
